# The influence of Dexmedetomidine as an additive to local anesthetics in dental procedures: A systematic review

**DOI:** 10.4317/medoral.26439

**Published:** 2025-01-26

**Authors:** Demóstenes Alves Diniz, Arthur José Barbosa de França, José Rodrigues Laureano-Filho, Eduardo Piza Pellizzer, Sandra Lúcia Dantas de Moraes, Belmiro Cavalcanti do Egito Vasconcelos

**Affiliations:** 1 M.Sc. student. Department of Oral and Maxillofacial Surgery, Faculty of Dentistry, University of Pernambuco (UPE), Recife, PE, Brazil; 2 Ph.D. student. Department of Oral and Maxillofacial Surgery, Faculty of Dentistry, University of Pernambuco (UPE), Recife, PE, Brazil; 3 Associate Professor. Department of Oral and Maxillofacial Surgery, Faculty of Dentistry, University of Pernambuco (UPE), Recife, PE, Brazil; 4 Full Professor. Department of Dental Materials and Prosthodontics, São Paulo State University (UNESP), Araçatuba, SP, Brazil; 5 Associate Professor. Division of Oral Rehabilitation, Faculty of Dentistry, University of Pernambuco (UPE), Recife, PE, Brazil; 6 Associate Professor. Department of Oral and Maxillofacial Surgery, Faculty of Dentistry, University of Pernambuco (UPE), Recife, PE, Brazil

## Abstract

**Background:**

This review aimed to assess if the use of dexmedetomidine as an additive to local anesthetics promotes greater safety and efficacy than local anesthetics alone in dental procedures.

**Material and Methods:**

the systematic review was structured according to the PICO strategy and adhered to the Preferred Reporting Items for Systematic Reviews and Meta-analyses (PRISMA) checklist. Studies were included based on the eligibility criteria, and data from the included studies were collected by one author. An additional author reviewed the compilation. Altogether, nine studies were included: eight randomized clinical trials and one controlled clinical trial. Of these, six were related to tooth extraction. Most studies reported the use of lidocaine as the local anesthetic. Levobupivacaine was used in one study.

**Results:**

In total, 352 patients were evaluated. Despite the heterogeneity between studies, it can be suggested that when used as an additive to local anesthetics, dexmedetomidine has the potential to decrease postoperative pain and latency period and prolong anesthetic duration.

Conclusion: There are scant reviews that summarizes the effectiveness of dexmedetomidine as an adjuvant to local anesthetics. These studies can positively impact the lives of the concerned population.

** Key words:**Anesthetics, adjuvants anesthesics, dexmedetomidine, oral surgery, systematic review.

## Introduction

Pain continues to remain a major concern in many patients despite significant advances in the field of pain management in dentistry (1). Local anesthetic solutions are routinely used in dental procedures to reduce pain using reversible nerve blocks (2,3). The choice of drug to be used as an anesthetic agent is based on three main considerations: potency, latency (time of onset of anesthesia), and duration of the anesthetic effect (4).

Adjuvants are co-administered with local anesthetic agents to improve the onset and/or duration of anesthesia (5). Epinephrine is one of the most commonly used adjuvants in combination with lidocaine or other anesthetic agents (6,7).

A new adjuvant, dexmedetomidine (DEX) has recently attracted the attention of researchers because it is a potent α-2 adrenoceptor agonist with sedative and analgesic actions (8). In literature, DEX has been proven to be 1620 times more selective for α-2 receptors and, in addition, it is associated with greater hemodynamic stability when compared to other adjuvants. The drug acts by binding to presynaptic α-adrenoreceptors, which results in the inhibition of epinephrine release. This leads to the termination of the propagation of pain signals (9).

However, recent clinical trials have focused on this new drug as a possible additive to local anesthesia. Studies suggest that the combination of DEX and lidocaine increases the nerve block time and decreases the onset of action. They also suggested that this association reduced pain scores to a significantly greater extent than lidocaine alone and decreased the frequency of postoperative analgesic use compared to the control group (5,7,9,10). The anti-inflammatory potential of DEX when injected locally promotes improved postoperative pain control (9,11).

There is a need for critical analysis of the results of these studies to present more clinically relevant data on this subject. In addition, there is a dearth of systematic reviews on this topic. Therefore, the current study was aimed at carrying out a systematic review and answer the following guiding question: “Does the use of DEX as an additive to local anesthetics promote greater safety and efficacy compared to local anesthetics without DEX in dental procedures?”.

Materials and Methods

- Registry Protocol

The present study adhered to Enhancing the Quality and Transparency of Health Research (EQUATOR network) recommendations, including the Preferred Reporting Items for Systematic Reviews and Meta-Analyses (PRISMA) (12,13) checklist. The methods used for this systematic review were recorded in the International Prospective Register of Systematic Reviews (PROSPERO - CRD 42022336235).

- Focused question

This systematic review was structured according to the PICO strategy: population, patients undergoing dental procedures under intraoral local anesthesia; intervention, intraoral local anesthesia with DEX; comparison, intraoral local anesthesia without DEX; main outcome, hemodynamic stability, onset, and duration of anesthesia; and additional outcome, pain during injection, postoperative pain, sedation, postoperative analgesics, blood loss, and patient satisfaction. The following focused question was proposed: “Does the use of DEX as an additive to local anesthetics promote greater safety and efficacy compared to local anesthetics without DEX in dental procedures?”

- Eligibility criteria

Studies were included based on the following criteria: randomized clinical trials and controlled clinical trials that compared local anesthetics with and without DEX in dental procedures. Pilot studies, investigations with no description of the solution anesthetics used, trials in which outcomes of interest were not available for analysis and the original values could not be retrieved after contacting the original authors, were excluded. Besides, the reports where the full-text articles were unavailable were also excluded. No data or language restrictions were applied.

- Data collection process

**Figure 1:**
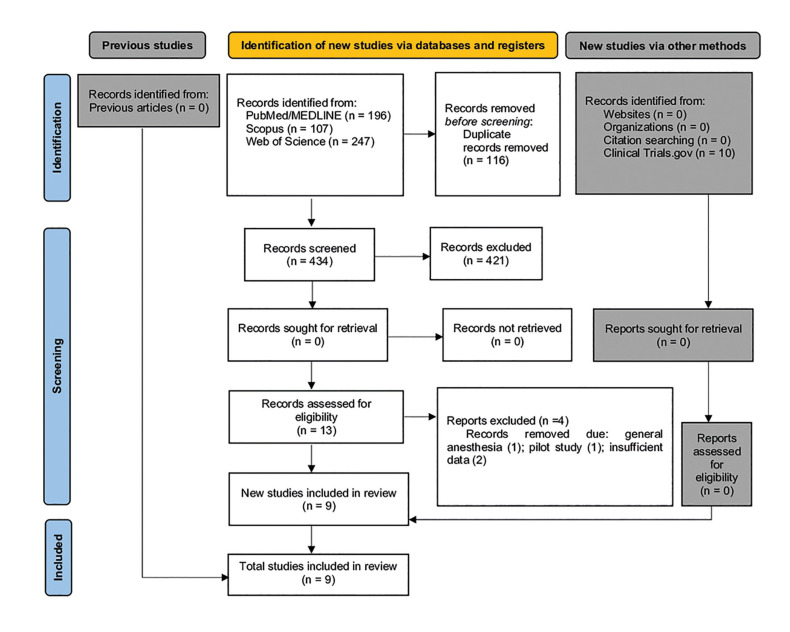
Flow diagram describing the studies selection.

First, the publications registered in all databases were imported to the Endnote Reference Manager to identify duplicate entries, as illustrated in the PRISMA flow diagram (Fig. 1). The selection process was conducted independently by two authors (DAD and AJBF) to examine the titles and abstracts of all identified publications by applying the inclusion criteria (blinded process). The same two reviewers applied the exclusion criteria, independently to the other studies. This was based on reading the full text (blinded process). The following data were collected from the articles: study design, age and samples, type of anesthetic, anesthetic volume, type of anesthetic adjuvant, anesthesia site, procedure performed, heart rate, diastolic and systolic blood pressure, oxygen saturation, time of onset of anesthesia, duration of anesthesia, pain during anesthetic injection/deposition, postoperative analgesics, level of sedation, blood loss, and patient satisfaction. The data collected from the articles are presented in the Tables.

- Additional analysis

Agreement between the investigators in determining study selection from each database search was evaluated using the kappa test, assuming an accepTable threshold value of 0.8 (14). Disagreements at any stage were resolved by discussion and mutual consensus with a third reviewer (BCEV).

- Search strategy

A search was conducted in the PubMed/MEDLINE, SCOPUS, Web of Science, and Clinical Trial.gov databases independently by two of the authors (DAD and AJBF) for articles published until April 30, 2023. The following terms were used in the search: (dexmedetomidine OR precedex) AND (local anesthesia OR dental anesthesia OR anesthesia infiltration OR nerve block OR nerve blockade OR levobupivacaine OR bupivacaine OR lidocaine OR lignocaine OR xylocaine OR prilocaine OR articaine OR mepivacaine OR ropivacaine) AND (dental OR dental care OR dental procedures OR dental surgical procedures OR dental surgery OR oral OR oral health OR oral procedures OR oral surgical procedures OR oral surgery OR tooth OR teeth OR impacted tooth OR impacted teeth OR third molar OR third molar surgery OR third molar extraction OR teeth extraction OR tooth extraction OR periodontitis OR periodontal disease OR periodontal surgery OR dental implants OR pulpitis OR endodontics). Any disagreements between the evaluators were resolved by a third author (BCEV) (Supplement 1).

The same authors performed a manual search for articles published in the following journals from January 2020 to June 2023: “Medicina Oral Patologia Oral y Cirurgia Bucal”, “International Journal of Oral and Maxillofacial Surgery”; “Journal of Oral and Maxillofacial Surgery”; “Journal of Cranio-Maxillofacial Surgery”; “British Journal of Oral and Maxillofacial Surgery”; “Journal of Clinical Anesthesia and Clinical Oral Investigations”. The search was also performed using ClinicalTrials.gov and the reference lists of the selected articles (gray literature) (Supplement 1).

- Risk of bias and quality assessment

**Figure 2:**
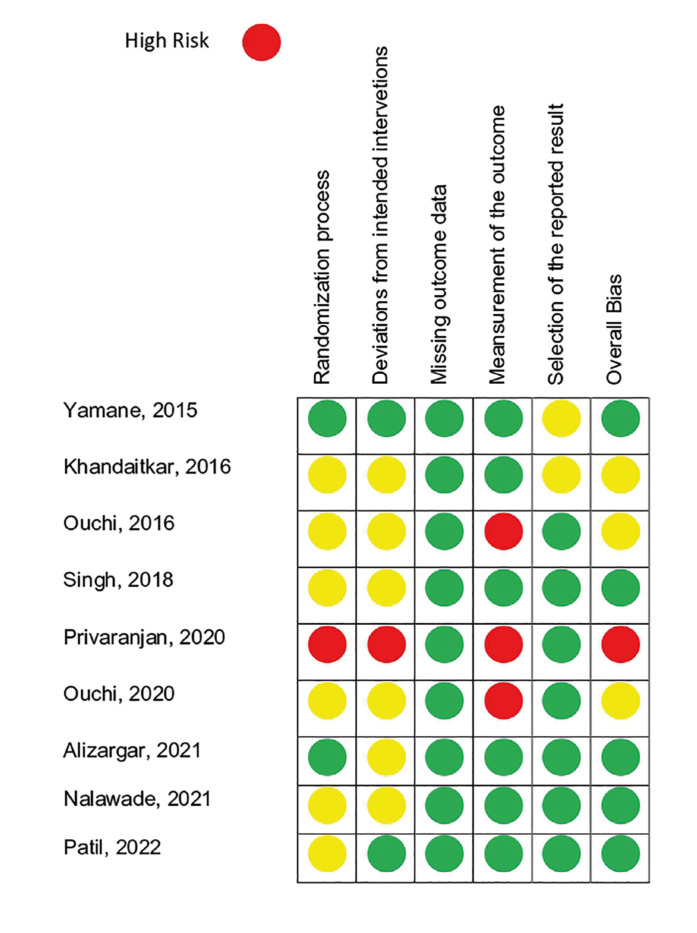
Assessment of the Risk Of Bias (ROB 2.0).

The article quality was evaluated using the Cochrane Risk of Bias tool (ROB 2.0) (15) for RCT studies. This tool evaluates selection bias (randomization process), performance bias (effect of assignment to intervention and mean outcome), bias detection, attrition bias (missing outcome data), reporting bias (selection of the reported result), and biases from other sources (Overall Bias) (Fig. 2). CCTs studies were evaluated using the Risk of Bias in Non-randomized Studies of Interventions (ROBINS-I) (12) (Supplement 2).

- Grading the body of evidence

For the final narrative synthesis, we used the Grading of Recommendations, Assessment, Development, and Evaluation (GRADE) to assess the certainty of evidence for narrative synthesis. This system classifies the quality of evidence into high, moderate, low, and very low, according to factors that consider the study design: risk of bias, inconsistency, indirectness, imprecision, and publication bias. The evidence was further assessed for dose-response, the effect’s magnitude, and residual confounders that could increase certainty (Supplement 3) (13,14). Studies were independently evaluated by two authors (DAD and AJBF), and agreement was reached in a consensus meeting with a third reviewer (BCEV), as required.

- Data extraction and synthesis of the results

Descriptive results were presented in the form of text, Figures, and Tables, in accordance with the PICO strategy. Data were evaluated based on the differences in mean and standard deviation between the intervention and control groups. The main and secondary outcomes were grouped and compared using an anesthetic solution (with and without DEX).

## Results

- Literature search

A total of 560 articles were retrieved from the PubMed/Medline (n = 196), Web of Science (n =247), Scopus (n =107), and Clinical Trial.gov (n =10) electronic databases. After removing duplicates (n =116), a manual search of the journals was performed. Titles and abstracts of the articles were read, and eligibility criteria were applied. A total of 13 articles underwent further analysis, from which four were excluded after reading the entire text. The exclusion criteria included: studies with patients undergoing general anesthesia, pilot studies and studies where no response was obtained from the authors about the data.

Finally, nine articles were included in the systematic review. A flowchart of the strategic research process has been shown in (Supplement 1). The Kappa score for the included studies retrieved from the respective databases was > 90%. This result suggested an “almost perfect agreement” between the examiners (14).

- Description of the studies

In Table 1, the qualitative characteristics of the nine included studies have been outlined. They are separated by the comparison of the control group (local anesthetic) and the DEX group (local anesthetic + DEX), eight randomized clinical trials, and one controlled clinical trial. Six studies (9,16-20) were related to tooth extraction. Other types of intervention were not reported by the author. A total of 382 patients were included in this study. In most studies, the local anesthetic used was lidocaine (8 of 9 studies) (9,11,16-18,20-22). The anesthetic was used in varying concentrations of 0.7%, 1%, 1.8%, and 2%. Levobupivacaine (0.5%) was used in only study (19). Seven studies used the inferior alveolar nerve block (9,11,16-21).

In Table 2, the quantitative characteristics of the included studies have been summarized. These which were separated by group comparison. The mean time of onset of anesthesia in the control group (3.53 min) was longer than that in the DEX group (3.36 min). The mean duration of anesthesia with lidocaine in the control group (141.05 min) was lower than that in the DEX group (189.47 min). Only one study analyzed the number of pills used for postoperative analgesia, with an average of 4.04 pills in the control group and higher than the average number in the DEX group (2.12 pills) (19).

In Table 3, the hemodynamic parameters found in some of the included studies during the period before (between 15 and 20 minutes), and after (from 60 minutes) surgery have been summarized. The mean heart rate was lower (79.82 bpm) in the DEX group than in the control group (80.78 bpm). Diastolic blood pressure in the control group showed an increased average (76.42 mmHg) compared to the DEX group (74.75 mmHg). Likewise, systolic blood pressure showed a lower value in the DEX group (116.46 mmHg/control:121.36). Oxygen saturation remained unchanged in both the groups. The parameters of pain, sedation level, and satisfaction after treatment are shown in Table 4. According to the visual analog scale (VAS), one of the included studies (19) showed that patients in the DEX group had 3.46 times lower postoperative pain scores. Two studies (16,19) assessed the degree of patient satisfaction and showed that a large number of patients considered the treatment good or excellent. Two studies (11,21) used the Mackenzie and Grant Sedation Score and no differences were observed between the two evaluated groups.

- Quality assessment of the studies

Cochrane Risk-of-Bias Tools (ROB 2.0) for Randomized Trials: A low risk of bias in “randomization process” was found in most of the studies, except for two (18,21) which had a moderate risk of bias. A low risk of bias was found in all studies for “Effect of assignment to intervention”, “Missing outcome data”, and “Measurements of the outcome”. Regarding the selection of reported results”, a moderate risk of bias was found in all studies. Overall, the selected studies were considered to have a low risk of bias (Fig. 2).

ROBINS-I: The only study that used this tool had a low risk of bias (20) (Supplement 2). Regarding the certainty of evidence, there was a low strength of evidence supporting the benefits of DEX for local anesthesia in dentistry and a low degree of recommendation for its use compared with traditional anesthetics.

- Certainty of evidence rating and strength of recommendations grading

In general, the onset of anesthesia, duration of anesthesia, pain during injection, blood loss, heart rate, diastolic blood pressure, systolic blood pressure, and oxygen saturation had a very low certainty of evidence, whereas postoperative analgesics, pain, sedation, and patient satisfaction had a low certainty of evidence.

## Discussion

Most of the included studies resulted in significant results. However, caution should be exercised when interpreting these results due to differences in assessments, little scientific strength with unclear methodologies in most of them. Although this systematic review retrieved a large number of RCTs, some data showed moderate bias, in addition to low or very low certainty of evidence. Limitations were found in the studies, including differences in dosages and reported concentrations of anesthetic solutions (1-2 mL and 0.5%-2%, respectively). Furthermore, the DEX concentration showed no standardization, ranging from 1.8 mcg to 180 mcg in the solutions (Table 1).

DEX is a promising drug, being the active dextro-isomer of medetomidine, which exhibits specific and selective α2-adrenoceptor agonism. Studies have shown that when used as an additive to local anesthetics, it shortens the latency period and prolongs its duration, corroborating the findings of the present study (Table 2). An important element of superiority was observed in terms of the duration time and faster onset of action in patients undergoing treatment with DEX in association with local anesthetic. The observed lower latency can be attributed to the blockade of presynaptic α2 receptors by DEX, which inhibits the release of norepinephrine, thereby ending the propagation of pain signals and prolonging hyperpolarization. This prevents the nerve from returning to the resting membrane potential (22).

The clinical action of DEX not only prolongs the duration of anesthesia, but also helps to reduce anxiety and induce sedation and analgesia (8). According to the parameters shown in Table 4, patients in the DEX group had considerably positive results from the point of view of postoperative pain according to the VAS (19). These results indicate the analgesic potency and prolonged duration of action of DEX, which can be attributed to its anti-inflammatory and local vasoconstriction effects, respectively. Studies have shown that DEX acts centrally, inhibiting the discharge of substance *P* by activating the α2 receptors at the locus coeruleus (20).

With respect to sedation (Table 4), two studies (11,21) presented results using the Mackenzie and Grant Sedation Scale. However, no differences were observed between the means of the evaluated groups. This result differs from those reported in literature (9,10,20), considering that DEX is a systemic sedative agent. Due to the limited number of studies and the studies being from the same author, caution should be exercised when interpreting these results.

In addition, hemodynamic stability seemed to be a positive factor in patients in the DEX group. According to the studies included in this review (Table 3), heart rate and systolic and diastolic blood pressure were lower ​​in the intraoperative period, reducing the chance of developing postoperative complications such as hemorrhages. This corroborated with occurrence of physiological processes related to hemostasis. When the blood pressure values are lower, the risk of bleeding in the intraoperative period is lower and lower is the rate of complications related to hemorrhage in the postoperative period. As observed in Table 2, one of the studies (20) showed 20% less bleeding in patients who used the solution with DEX. However, it is important to note that only one study evaluated the blood loss variable, which becomes an important limitation in the analysis of the outcome. Maintenance of oxygen saturation was also observed in both groups (Table 3). The administration of DEX did not cause respiratory depression, thereby not affecting the respiratory system or tissue oxygen perfusion.

Local anesthetics have inverted kinetics: they first exert a local action on the tissue and then are distributed and biotransformed in the body, unlike systemic use. Therefore, the objective of DEX as an additive is to evaluate the possible benefits associated with the local anesthetic already demonstrated in the primary studies.

In local anesthetics currently used in dentistry, vasoconstrictors are added, such as epinephrine, which in medicine is commonly found in 1:1000 ampoules. The concentrations used in dental anesthetic tubes are normally 1:100,000 or 1:200,000, which guarantee greater safety and allow for cautious use. DEX as an adjuvant to local anesthetics has the same objective. Studies are being carried out to find an ideal concentration that combines efficacy and safety for use in Dentistry. Its concentration is not the same as that used in medicine, as the purpose is different. In Dentistry, they are not being studied for the purpose of conscious sedation and general anesthesia.

Information on side effects as an anesthetic adjuvant is scarce or not observed, as the quantity used in studies is limited, with no possibility of overdose. We believe that as new studies emerge, complications can be better understood. Thus, the present work does not aim to support the immediate use of DEX as an additive, but to bring the existing results in the literature and also to support future studies, so that it may become an alternative to conventional anesthetics with vasoconstrictors.

Due to the heterogeneity in the types, dosages, and concentrations of local anesthetics, it was not possible to perform a meta-analysis in this systematic review. Therefore, qualitative analysis of the data was conducted. Despite this, DEX has important properties and potential for future studies in the field of Local Anesthesiology in Dentistry.

## Figures and Tables

**Table 1:** General data of the studies, anesthetics with and without DEX, procedures performed and anesthesia site.

Author/year of publication	Study design	Age/sample	Volume/Local Anesthetic (control)	Volume/Local Anesthetic (DEX)	Procedure performed	Anesthesia site
Yamane, 2015	RCT	25-32 / 20	1ml Lidocaine 0.7%*	1ml Lidocaine 0.7% + 1mmol/L DEX*	NR	Infiltration (anterior teeth)
Khandaitkar, 2016	RCT	> 18 / 90	2ml Lidocaine 2% + 0.14ml SS	2ml Lidocaine 2% + 0.14ml DEX	Extraction	IONB
Ouchi, 2016	RCT	21-32 / 18	Lidocaine 1% + ep. 1:80.000	1. Lidocaine 1% + 2.5ppm DEX 2. Lidocaine 1% + 5.0ppm DEX 3. Lidocaine 1% + 7.5ppm DEX	NR	IANB
Singh, 2018	RCT	10-25 / 25	Lidocaine 2% + ep. 1:200.000	Lidocaine 2% + 30mg DEX	Extraction	IONB, IANB, GP, LNB
Priyaranjan, 2020	RCT	20-30 / 40	Lidocaine 2% + ep. 1:80.000	Lidocaine 2% + 1μ/ml DEX	Extraction of third molar	IANB, LNB
Ouchi, 2020	RCT	NR / 19	Lidocaine 1,8% + ep. 1:80.000	1. 1.8ml Lidocaine 1.8% + 1.0ppm DEX 2. 1.8ml Lidocaine 1.8% + 2.0ppm DEX	NR	IANB
Alizargar, 2021	RCT	18-40 / 40	Lidocaine	Lidocaine + 15ml DEX	Extraction of third molar	IANB
Nalawade, 2021	RCT	18-35 / 80	Lidocaine 2% + ep. 1:200.000	Lidocaine 2% + DEX 1mcg/ml of Lidocaine	Extraction of third molar	IANB, BNB
Patil, 2022	RCT	22-48 / 50	1.8ml Levobupivacaine 0.5% + 0.2ml SS	1.8ml Levobupivacaine 0.5% + 0.2ml DEX	Extraction of third molar	IANB

DEX: Dexmedetomidine, RCT: Randomized clinical trial, CCT: Controlled clinical trial, ml: milliliters, mg: milligrams, %: percent, mmol/L: Milimol per liter, ep: epinephrine, SS: Saline Solution, ppm: parts per million, mcg: Microgram, μ: Micro, IONB: Infraorbital nerve block, IANB: Inferior alveolar nerve block, LNB: Lingual nerve block, BNB: Buccal nerve block, GP: Greater palatine; NR: not reported; *at a final concentration of 0.7%.

**Table 2:** Onset time and duration of anesthesia, injection pain, use of analgesics and blood loss.

Author/ year of publication	Onset time		Duration		Pain during injection				Postoperative analgesics		Blood loss	
	C	I	C	I	C	I			C	I	C	I
Khandaitkar, 2016	261.67 s	185 s	70.43 s	124.87 s	NR	NR			NR	NR	NR	NR
Ouchi, 2016	40020 s	Dex 2.5: 333.6 s Dex 5.0: 267 s Dex 7.5: 250.2 s	131220 s	Dex 2.5: 142380 s Dex 5.0: 157440 s Dex 7.5: 173460 s	PL: 11 MI: 4 MO: 2 SE: 1	Dex 2.5 PL: 7 MI: 6 MO: 5 SE: 0	Dex 5.0 PL: 6 MI: 6 MO: 6 SE: 0	Dex 7.5 PL: 7 MI: 6 MO: 5 SE: 0	NR	NR	NR	NR
Singh, 2018	Mand: 141 s	Mand: 113 s	Mand: 9420 s	Mand: 10440 s	NR	NR			NR	NR	NR	NR
Priyaranjan, 2020	95 s	138 s	4200 s	7920 s	NR	NR			NR	NR	10 ml	8 ml
Ouchi, 2020	2460 s	Dex 1.0: 2580 s Dex 2.0: 2760 s	147300 s	Dex 1.0: 130620 s Dex 2.0: 142860 s	PL: 0 MI: 11 MO: 8 SE: 0	Dex 1.0 PL: 1 MI: 10 MO: 7 SE: 1	Dex 2.0 PL: 1 MI: 9 MO: 9 SE: 0		NR	NR	NR	NR
Nalawade, 2021	11160 s	5340 s	46020 s	59040 s	NR	NR			NR	NR	NR	NR
Patil, 2022	203.6 s	119.9 s	292320 s	465840 s	NR	NR			4.04 ps	2.12 ps	NR	NR

C: control, DEX: dexmedetomidine, I: Intervention, Mand: mandible, MI: mild, ml: milliliters, MO: moderate, NR: not reported, PL: painless, ps: pills, s: seconds, SE: severe.

**Table 3:** Hemodynamic data from the included studies.

Author/year of publication			Yamane, 2015	Singh, 2018	Priyaranjan, 2020	Alizargar, 2021	Patil, 2022	Nalawade, 2021
Heart rate (bpm)	Control	Pre	69.3	78.9	84	84.4	74.24	76.58
		Trans	70.1	97.2	97	84.95	75.36	79.25
		Post	66.8	82.8	85	84.55	76.8	79.1
	Intervention	Pre	71.9	81	84	86.75	75.2	79.7
		Trans	71.1	94.6	73	90.50	73.84	78.9
		Post	71.7	86.2	83	89.55	77.68	77.13
Diastolic blood pressure (mmHg)	Control	Pre	63.3	71.1	81	72	74.04	81.13
		Trans	66.5	70.4	82	74.5	74.48	82.25
		Post	66.1	70.4	80	73	73.04	83.5
	Intervention	Pre	65.8	71.6	81	75.5	75.36	81.75
		Trans	67.6	70.5	74	77	73.12	80.75
		Post	69.2	70.5	80	75	71.84	81.25
Systolic blood pressure (mmHg)	Control	Pre	114.4	117.5	122	NR	113.36	125.6
		Trans	111.5	117.6	135		113.68	114.1
		Post	115.6	117.2	120		115.28	133
	Intervention	Pre	113.7	116.9	122		119.2	127.5
		Trans	116.0	115.8	110		116.48	117.9
		Post	114.1	116	121		116.8	118
Oxigen saturation (%)	Control	Pre	NR	99.9	NR	97.8	98.2	97.42
		Trans		99.9		97.45	98.2	97.25
		Post		100		97.20	98.12	97.62
	Intervention	Pre		99.9		97.15	97.96	97.5
		Trans		99.9		96.85	98.2	98.57
		Post		100		96.55	98.28	99.02

bpm: beats per minute, mmHG: millimeters of mercury, %: percent, pre: preoperative, trans: transoperative (between 15 to 20 minutes), post: postoperative (from 60 minutes), NR: not reported.

**Table 4:** Pain, sedation and satisfaction.

Author/year of publication	Pain		Sedation		Patient satisfaction	
	Control	Intervention	Control	Intervention	Control	Intervention
Yamane, 2015	927m (CPT)	1039m (CPT)	NR	NR	NR	NR
Ouchi, 2016	NR	NR	Mackenzie and grant sedation score (1-5) 10 minutes Level 1: 17 Patients Level 2: 1 Patient 60 minutes Level 1: 17 Patients Level 2: 1 Patient	Mackenzie and grant sedation score (1-5) 10 minutes Dex 2.5% Level 1: 18 Patients Dex 5.0% Level 1: 17 Patients Level 2: 1 Patient Dex 7.5% Level 1: 18 Patients 60 minutes Dex 2.5% Level 1: 18 Patients Dex 5.0% Level 1: 14 Patients Level 2: 3 Patients Level 3: 1 Patient Dex 7.5% Level 1: 17 Patients Level 2: 1 Patient	NR	NR
Ouchi, 2020	NR	NR	Mackenzie and grant sedation score (1-5) 10 minutes Level 1: 19 Patients 60 minutes Level 1: 18 Patients Level 2: 1 Patient	Mackenzie and grant sedation score (1-5) 10 minutes Dex 1.0 ppm Level 1: 19 Patients Dex 2.0 ppm Level 1: 18 Patients Level 2: 1 Patient 60 Minutes Dex 1.0 ppm Level 1: 18 Patients Level 2: 1 Patient Dex 2.0 ppm Level 1: 16 Patients Level 2: 1 Patient	NR	NR
Alizargar, 2021	NR	NR	NR	NR	High: 16 Patients (80%) Intermediate: 1 Patient (20%)	High: 19 Patients (95%) Intermediate: 1 Patient (5%)
Patil, 2022	6h: 1.8 (VAS) 12h: 2.7 (VAS) 24h: 3.7 (VAS) 48h: 3.7 (VAS) 72h: 3.7 (VAS)	6h: 0 (VAS) 12h: 0.3 (VAS) 24h: 1.0 (VAS) 48h: 1.6 (VAS) 72h: 1.6 (VAS)	NR	NR	1. Bad: 3 (7.89%) 2. Moderate: 8 (21.05%) 3. Good: 14 (36.84%) 4. Excellent: 13 (34.21%)	1. Bad: 1 (2.63) 2. Moderate: 5 (13.15) 3. Good: 10 (26.31) 4. Excellent: 22 (57.89)

NR: not reported, m: minutes, CPT: Current Perception Threshold, %: percent, h: hour, VAS: Visual Analogic Scale, ppm: parts per million, DEX: Dexmedetomidine.
